# Tissue factor pathway inhibitor upregulates CXCR7 expression and enhances CXCL12-mediated migration in chronic lymphocytic leukemia

**DOI:** 10.1038/s41598-021-84695-8

**Published:** 2021-03-04

**Authors:** Xue Yan Cui, Geir Erland Tjønnfjord, Sandip M. Kanse, Anders Erik Astrup Dahm, Nina Iversen, Christiane Filion Myklebust, Ling Sun, Zhong Xing Jiang, Thor Ueland, James J. Campbell, Mitchell Ho, Per Morten Sandset

**Affiliations:** 1 Department of Haematology, The First Affiliated Hospital of Zhengzhou University, No. 1 Jianshe East Road, Zhengzhou, 450000 China; 2 Department of Haematology, Oslo University Hospital Rikshospitalet, Nydalen, Box 4950, 0424 Oslo, Norway; 3 Research Institute of Internal Medicine, Oslo University Hospital, Oslo, Norway; 4 Institute of Clinical Medicine, University of Oslo, Oslo, Norway; 5 K.G. Jebsen Centre for B-Cell Malignancies, University of Oslo, Oslo, Norway; 6 Institute of Basal Medical Sciences, University of Oslo, Oslo, Norway; 7 Department of Haematology, Akershus University Hospital, Lørenskog, Norway; 8 Department of Medical Genetics, Oslo University Hospital, Oslo, Norway; 9 ChemoCentryx Headquarters, Mountain View, CA USA; 10 Laboratory of Molecular Biology, Center for Cancer Research, National Cancer Institute, National Institutes of Health, Bethesda, USA

**Keywords:** Biomarkers, Risk factors

## Abstract

The infiltration of chronic lymphocytic leukemia (CLL) cells into lymphoid organs correlates with disease severity. CXCL12 is a key chemotactic factor for the trafficking of CLL. Tissue factor pathway inhibitor (TFPI) is a serine protease inhibitor and plays a role in CXCL12-mediated hematopoietic stem cell homing. We aim to explore the role of TFPI in CXCL12-mediated migration of CLL cells. In this study, plasma TFPI concentrations were measured by ELISA. CLL cells were isolated from patients and used for trans-endothelial migration (TEM) assays. Quantitative RT-PCR and Western blotting were used to detect the expression of CXCR7, CXCR4 and β-catenin. Immunofluorescence and co-immunoprecipitation was used to detect the binding of TFPI and glypican-3 (GPC3). We found that plasma TFPI levels in CLL patients were higher than in healthy controls, particularly in the patients with advanced disease. TFPI enhanced CXCL12-mediated TEM of CLL cells by increasing the expression of the CXCL12 receptor CXCR7, but not of the CXCL12 receptor CXCR4. The effect of TFPI on TEM was abolished by the CXCR7 inhibitor, CCX771, while the CXCR4 inhibitor AMD3100 strongly increased TEM. TFPI co-localized with GPC3 on the cell surface. An antibody to GPC3, HS20, decreased CXCR7 expression and abolished the effect of TFPI on TEM. TFPI activated β-catenin and the Wnt/β-catenin inhibitor IWP4 repressed the effect of TFPI on CXCR7 expression and TEM. We conclude that TFPI may contribute to organ infiltration in CLL patients.

## Introduction

With an age-adjusted incidence of 4–5 per 100,000 per year chronic lymphocytic leukemia (CLL) is the most common type of leukemia in the western world^1^. Infiltration of CLL cells in lymphoid tissues is a key element of the disease pathogenesis^2^. CLL cells infiltrate primary and secondary lymphoid organs where they are protected from apoptosis through crosstalk with stromal cells^3^. Critically, CLL cells in lymphoid niches are protected against cytotoxic effects of many chemotherapeutics and likely cause minimal residual disease and future relapse^4^. Growing evidence indicates that lymphocyte trafficking plays a critical role in the pathophysiology of CLL^5^. Interfering with CLL cell migration or retention in lymphoid tissues could thus improve the efficacy of conventional therapy of CLL patients.

The C-X-C motif chemokine ligand 12 (CXCL12), also called chemokine stromal-derived factor 1 (SDF1α), is constitutively secreted by fibroblasts and stromal cells and attracts leukemic cells to the specific microenvironment necessary for their survival^6^. A recent study showed increased levels of CXCL12 in serum of symptomatic late stage CLL patients in comparison to patients with early stage CLL^7^. The chemokine receptor CXCR4 was the first identified receptor for CXCL12^8^, and CXCR7, also known as RDC1 or atypical chemokine receptor 3 (ACKR3), was recently reported as a receptor for CXCL12^9^. CXCR7 has a stronger affinity for CXCL12 than CXCR4^10^, and ligand binding to CXCR7 activates alternative signaling pathways regulating cellular adhesion, proliferation and dissemination of tumor cells^11^ as well as differentiation of mature B cells^12^. Furthermore, CXCR7 has been shown to potentiate and regulate trans-endothelial migration (TEM) of circulating tumor cells leading to enhanced extravasation^13^. However, the role of CXCR7 in CLL cell trafficking has not been elucidated, and further understanding of this receptor may facilitate the development of novel therapies.

CLL cell homing involves cell adhesion molecules and chemotactic factors which are highly influenced by endothelial cells^14^. It is well known that endothelial cells are the main source of tissue factor (TF) pathway inhibitor (TFPI), which is the primary inhibitor of the initiation of blood coagulation and inhibits both TF-factor (F) VIIa-dependent FXa generation and free FXa^15^. The interaction between TF-FVIIa and TFPI promote tumor cell adhesion and migration^16^. TFPI inhibits primary and metastatic tumor growth and represses endothelial proliferation in vitro^17^. We have previously reported that TFPI is involved in cell migration in breast cancer^18^.

A recent study showed that TFPI increases hematopoietic stem cell homing by binding to glypican-3 (GPC3), a heparan sulfate proteoglycan^19^, and regulates the activity of CXCL12^20^. GPC3 regulates canonical Wnt/β-catenin signaling^21^, which is activated in hematopoietic malignancies and contributes to tumor recurrence^22^. The activation of Wnt/β-catenin leads to the accumulation of β-catenin and favors its translocation to the nucleus as a cofactor activating the transcription of Wnt/β-catenin target genes^23^.

We investigated the role of TFPI in the trans-endothelial migration of CLL cells. The effect of TFPI was analyzed in detail with respect to regulation of the key chemotactic factor CXCL12 and its receptors. The role of the potential TFPI receptor, GPC3, and the involvement of the Wnt/β-catenin signaling pathway were examined. We have unraveled a novel mechanism of action of TFPI that correlates with its higher levels in patients with advanced stage CLL.

## Materials and methods

### Human samples, cell lines and reagents

All CLL patients and healthy controls provided written informed consent using protocols approved by the Regional Committee for Medical and Health Research Ethics (Approval No. 2016/947/REC South-East A) and in accordance with the principles of the Declaration of Helsinki. Patients were diagnosed according to the International Workshop on Chronic Lymphocytic Leukemia 2008 criteria^24^.

To obtain peripheral blood mononuclear cells (PBMCs), heparinized blood samples from CLL patients and buffy coats obtained from healthy donors at the Blood Transfusion Centre of the Oslo University Hospital were centrifuged using Lymphoprep (Alere Technologies AS, Oslo, Norway) in SepMate 50 mL tubes (Stem Cell Technologies, Cambridge, UK). CLL and normal B cells were purified from the PBMCs by negative selection of MACS B cell Isolation Kit II (Miltenyi Biotec, Auburn, CA, USA) by using AutoMACS cell separator (Miltenyi Biotec, Bergisch Gladbach, Germany). The amount of antibodies and beads was reduced to 1/10 for CLL B cell isolation. The purities of CLL or normal B cell enrichments were 95% or greater, determined by flow cytometry analysis staining for CD19+ cells. The purified CLL cells and normal B cells were incubated in RPMI1640 medium (Lonza, Verviers, Belgium) supplemented with 10% fetal bovine serum (FBS) (Lonza), 100 U/mL penicillin and 100 µg/mL streptomycin (Lonza).

The CLL cell line HG3 (DSMZ-German Collection of Microorganisms and Cell Cultures, Braunschweig, Germany) and the chronic myeloid leukemia blast crisis cell line K562 provided by the Institute of Hematology of the Chinese Academy of Medical Sciences (Tianjin, China) were grown in flasks containing RPMI1640 medium (Lonza) supplemented with 10% FBS (Lonza), 100 U/mL penicillin and 100 µg/mL streptomycin (Lonza).

Human umbilical vein endothelial cells (HUVECs, CC-2517, Lonza) were grown in complete endothelial cell growth medium MV2 (Promocell, Heidelberg, Germany).

Human recombinant TFPI (rTFPI) and human recombinant CXCL12 (rCXCL12) were obtained from R&D Systems (Minneapolis, MN, USA). CCX771, a novel specific CXCR7 inhibitor was provided by ChemoCentryx (Mountain View, CA, USA). AMD3100, a specific inhibitor for CXCR4 was obtained from Merck KgaA (Darmstadt, Germany). HS20, a human monoclonal antibody against the heparan sulphate chains of GPC3^25^ was kindly provided by Dr. Mitchell Ho, U.S.National Cancer Institute (NCI). IWP4, a potent inhibitor of Wnt/β-catenin signaling was obtained from Tocris Bioscience (Bristol, UK).

### Free TFPI ELISA antigen detection

Citrated venous blood samples were collected from 36 CLL patients (25 males and 11 females, mean age 65 years, range 38–80 years). Patients were also clinically staged according to the Binet staging system^26,27^ with the following distribution: stage A, 21 (58.3%), stage B, 7 (19.4%) and stage C, 8 (22.2%). Citrated venous blood samples from 34 healthy subjects (17 males and 17 females, mean age 42 years, range 24–68 years) were used as controls. Plasma samples were collected by centrifugation at 2500×*g* for 15 min at 20 °C. Plasma aliquots were frozen and stored at − 80 °C until being assayed. The commercial enzyme-linked immunosorbent assay (ELISA) Asserachrom Free TFPI (Diagnostica Stago, Asnières, France) was used to measure the concentration of full-length TFPI in the plasma according to the manufacturer’s protocol^28^.

### Transendothelial migration (TEM) assays

TEM assays were performed in transwell insert plates (96-well, 3 µm pore size; Costar, Corning). Approximately 30 000 HUVECs were seeded into the transwell the day before the chemotaxis assay and incubated overnight to generate a HUVEC monolayer. Fresh CLL or normal B cells were incubated in RPMI1640 medium with 10% FBS and treated with rTFPI at the indicated concentrations for 24 h before the migration assays. In some experiments, the CLL cells were pre-treated with HS20 or IWP4 for 1 h prior to rTFPI treatment. Then the cells were washed with RPMI1640 without serum and 3.75 × 10^5^ CLL cells or normal B cells were resuspended in 75 µL RPMI1640 supplemented with 1% BSA (Sigma Aldrich, MO, USA) and placed in the upper chamber of transwell inserts. Inserts were placed in the wells containing 235 µL medium alone (basal) or medium with 400 ng/mL rCXCL12. Stimuli were applied at optimal concentrations determined by previous titration. Plates were centrifuged shortly (for 1 s at 150 g) to spin down the cells onto the filter and migration proceeded for 3 h in the incubator (37 °C, 5% CO_2_). Migrated cells were harvested from the lower chamber and counted by flow cytometer MACS Quant (Miltenyi Biotec GMbH, Bergisch Gladbach, Germany). Cell migration capacity was expressed either as the percentage of migrated cells, or as a fold change, which is defined as the number of migrated cells in the presence of rCXCL12 divided by the number of migrated cells in the absence of rCXCL12.

### Western blot analysis

Protein extracts were prepared as previously described^29^. 20–50 µg proteins were resolved by SDS-PAGE, transferred to polyvinylidene difluoride (PVDF) membranes (Bio-Rad Laboratories, Hercules, CA, USA). Then the PVDF membranes were cut along the 75 kDa level or between 75 and 50 kDa according to the protein standards (Bio-Rad Laboratories). The upper parts of the membranes were incubated with the antibodies against active β-catenin (Cell Signaling Technology, Boston, MA, USA) or β-catenin (Novus Biologicals); the lower parts of the membranes were incubated with the antibodies against CXCR7 (Novus Biologicals, Centennial, CO, USA), CXCR4 (Abcam, Cambridge, UK) or β-actin (Cell Signaling Technology). Detections were performed as previously described^29^.

### Flow cytometry

CLL cells were isolated from CLL patients and treated with 200 ng/mL rTFPI for 24 h. Then the cells were washed and blocked by human FcR blocking reagent (Miltenyi Biotec, Bergisch Gladbach, Germany) for 10 min at room temperature. Afterwards, the cells were stained with PE-conjugated anti-human CXCR7 antibody (Biolegend, San Diego, CA) for 1 h. PE-conjugated mouse IgG2b was used as isotype control (Biolegend). The expression of CXCR7 was measured by a FACS Calibur flow cytometry (Becton Dickinson, Franklin Lakes, NJ, USA) and analyses were carried out using FlowJo software (Becton Dickinson).

### RNA isolation, cDNA synthesis and relative quantification of mRNA

Total RNA was extracted, quantified and reversely transcribed to cDNA as described before^30^. Quantitative RT-PCR was used to measure the relative mRNA expression of CXCR7 (ACKR3, Hs00604567_m1, Applied Biosystems) and CXCR4 (Hs00237052_m1, Applied Biosystems). Ct values were normalized against the endogenous control TBP (Applied Biosystems). Negative controls without cDNA were always included.

### Immunofluorescence staining and confocal microscopy studies

Expression of TFPI and GPC3 in HG3 cells before or after rTFPI treatment was examined by immunostaining. 20 µL cell suspension were spread on a slide and air-dried for 10 min. Cells were fixed by ethanol for 10 min at 4 °C and incubated with anti-TFPI (ADG72, Sekisui Diagnostics, Pfungstadt, Germany) and anti-GPC3 (1G12, Santa Cruz, CA, USA) primary antibodies overnight at 4 °C. Normal rabbit IgG (Cell Signaling Technology) and mouse IgG1 (R&D Systems Inc., MN, USA) were used as controls. Following washing with Dulbecco’s phosphate buffered saline (Sigma Aldrich, MO, USA) in 2% fetal calf serum, cells were stained with Alexa Fluor 488-labeled donkey anti-rabbit and Alexa Fluor 568-labeled donkey anti-mouse secondary antibodies (Thermo Fisher Scientific, Eugene, OR, USA) for 90 min at room temperature. Nuclei were stained with diamidino-2-phenylindole (ProLong Gold antifade reagent, Thermo Fisher Scientific). Fluorescence images were acquired by using a Leica TCS SP8 confocal microscope equipped with a 40 × 1.30 NA oil immersion lens, a UV (405 nm) laser and a CW (Continuous Wavelength) white light laser. Sequential acquisition scanning was carried out at 1024 × 1024 pixel resolution. For each of the cells, 10–20 Z-scans spanning the entire cell were generated. Z-stack images were acquired by using ImageJ software.

### Co-immunoprecipitation

The total protein content of HG3 and K562 cell lysate was measured with the Pierce BCA protein assay Kit (Thermo Scientific Pierce, Rockford, IL, USA). 3 mg of total proteins from HG3 and K562 cells were used for co-immunoprecipitation (Co-IP) experiments. Co-IP was performed using the Co-IP Kit (Thermo Scientific Pierce) following the manufacturer’s protocol. Briefly, 20 mg of TFPI antibody (MAB2974, R&D Systems Inc., MN, USA) and isotype control mouse IgG2α (R&D systems) were incubated with AminoLink Plus coupling resin for 2 h at room temperature. The antibody-coupled resins were incubated with 500 µL of the whole protein lysates overnight at 4 °C. The resins were washed and the protein complexes bound to the antibody were eluted. Subsequent Western blot analysis was done as described before^29^. The pulling down of GPC3 was detected by using sheep anti-GPC3 (AF2119, R&D systems). The detection of TFPI with goat anti-TFPI antibody (AF2974, R&D systems) was used as a control for IP.

### Statistics

We performed statistical analysis using Prism (version 8.0.1, Graphpad software, San Diego, CA, USA) and SPSS version 25.0.0.1. For in vitro data, mean ± SEM of n = 3 or more determinations are shown. We used two-tailed Student’s t test or one-way ANOVA test. For comparison of plasma TFPI levels, TFPI values were slightly skewed as evaluated by the Kolmogorov Smirnov test and were log transformed prior to UNIANOVA, which was age and Bonferroni-adjusted. Differences were considered statistically significant at a two-tailed value of P < 0.05.

## Results

### Higher plasma TFPI concentration was detected in CLL patients with advanced disease

**Figure 1:**
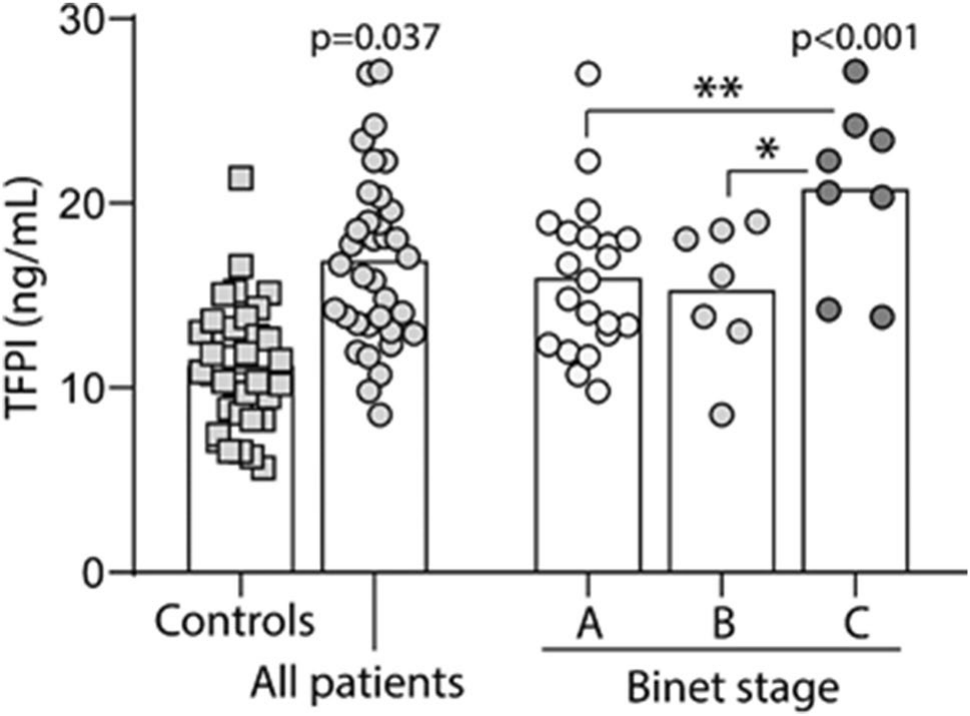
Plasma TFPI concentration increased in patients with CLL and related to CLL progression. Increased plasma TFPI level was observed in CLL patients compared to healthy controls. Compared to the patients with Binet stage A and B, the patients with Binet stage C showed the highest TFPI concentration in the plasma. Binet stages represent the severity of organ infiltration. Data are presented as dot plots and columns represent median levels. Concentrations are expressed as ng/mL. p values in the graph represent differences vs. controls. *p < 0.05, **p < 0.01. All statistical comparisons were adjusted for age using UNIANOVA with Bonferroni adjustment on log transformed TFPI values.

The main characteristics of all the untreated CLL patients are described in Table 1. CLL patients exhibited elevated TFPI levels (median: 16.9 ng/mL, range: 8.5–27.2 ng/mL) as compared to healthy controls (median: 10.8 ng/mL, range: 5.7–21.4 ng/mL; Fig. 1). No differences in sex distribution between patients and controls were detected, but patients were older and age was used in adjustment of p-values. To address the severity of CLL, we applied the Binet staging system which relates to organ infiltration. The results showed that patients with Binet stage C (median: 21.5 ng/mL, range: 13.8–27.2 ng/mL) had higher plasma TFPI level than patients with Binet stage A (median: 15.8 ng/mL, range: 9.8–27.0 ng/mL) and Binet stage B (median: 16.1 ng/mL, range: 8.5–19.0 ng/mL) (Fig. 1), suggesting that higher plasma TFPI relates to the severity of organ infiltration.

**Table 1:** Characteristics of all the untreated CLL patients stratified according to Binet stages.

Variables	All patients (n = 36)	Binet stage A (n = 21)	Binet stage B (n = 7)	Binet stage C (n = 8)
Age (> 65 years)	18	11	2	5
Gender (M/F)	25/11	14/7	6/1	5/3
Homology with germ line (≥ 96%)	10	3	2	5
Lymphocytes (> 50 × 10^9^/L)	15	3	6	6
Hemoglobin (< 10 g/dL)	4	0	0	4
Platelet (< 100 × 10^9^/L)	2	0	0	2

### TFPI enhanced CXCL12-mediated TEM of CLL cells

**Figure 2:**
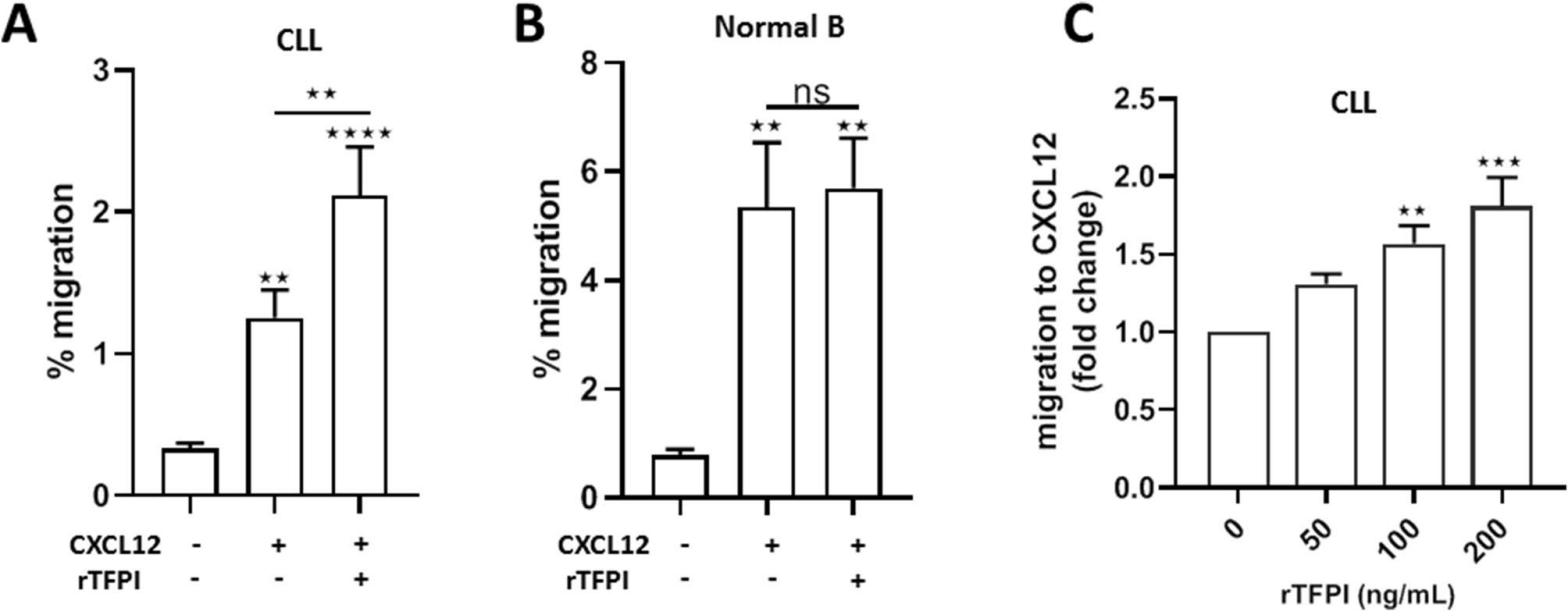
TFPI enhances CXCL12-mediated TEM of CLL cells, but not normal B cells. (**A**) Fresh CLL cells were pre-treated with or without 200 ng/mL rTFPI for 24 h and then washed and applied to the TEM assay for 3 h with or without 400 ng/mL recombinant CXCL12 added to the lower chamber. The percentage was determined by counting the migrated fraction of the input cells. Data are presented as mean ± SEM; n = 8 individuals, **P < 0.01, ****P < 0.0001. (**B**) Normal B cells were also treated with or without rTFPI and used in the TEM assay as described above. Data are presented as mean ± SEM; n = 4 individuals, **P < 0.01. (**C**) Fresh CLL cells were treated with 0, 50, 100 and 200 ng/mL rTFPI for 24 h and then washed and applied to the TEM assay as described above. The number of migrated cells in the lower chamber was counted and cell migration to CXCL12 was expressed as fold change relative to control. Data are presented as mean ± SEM; n = 5 individuals, **P < 0.01, ***P < 0.001.

To explore the possible role of TFPI in organ infiltration of CLL patients, we first investigated whether rTFPI treatment affects the migration of CLL cells in response to CXCL12 compared to normal B cells. After exposure to exogenous rTFPI, CLL or normal B cells were applied to the TEM assay. Results showed that CXCL12 significantly induced TEM of CLL (Fig. 2A) and normal B cells (Fig. 2B). These findings indicated that CXCL12 was an efficient chemoattractant for both CLL and normal B cells. However, rTFPI significantly enhanced the CXCL12-mediated TEM of CLL cells (Fig. 2A), while no effect of rTFPI was found on normal B cells (Fig. 2B). Furthermore, in CLL cells, the CXCL12-mediated migration was increased by rTFPI in a dose-dependent manner (Fig. 2C). We concluded that TFPI enhanced the CXCL12-mediated TEM of CLL cells but had no effect on normal B cells despite of their higher migration capacity in response to CXCL12.

### TFPI upregulated the expression of receptor CXCR7, but not CXCR4 in CLL cells

**Figure 3:**
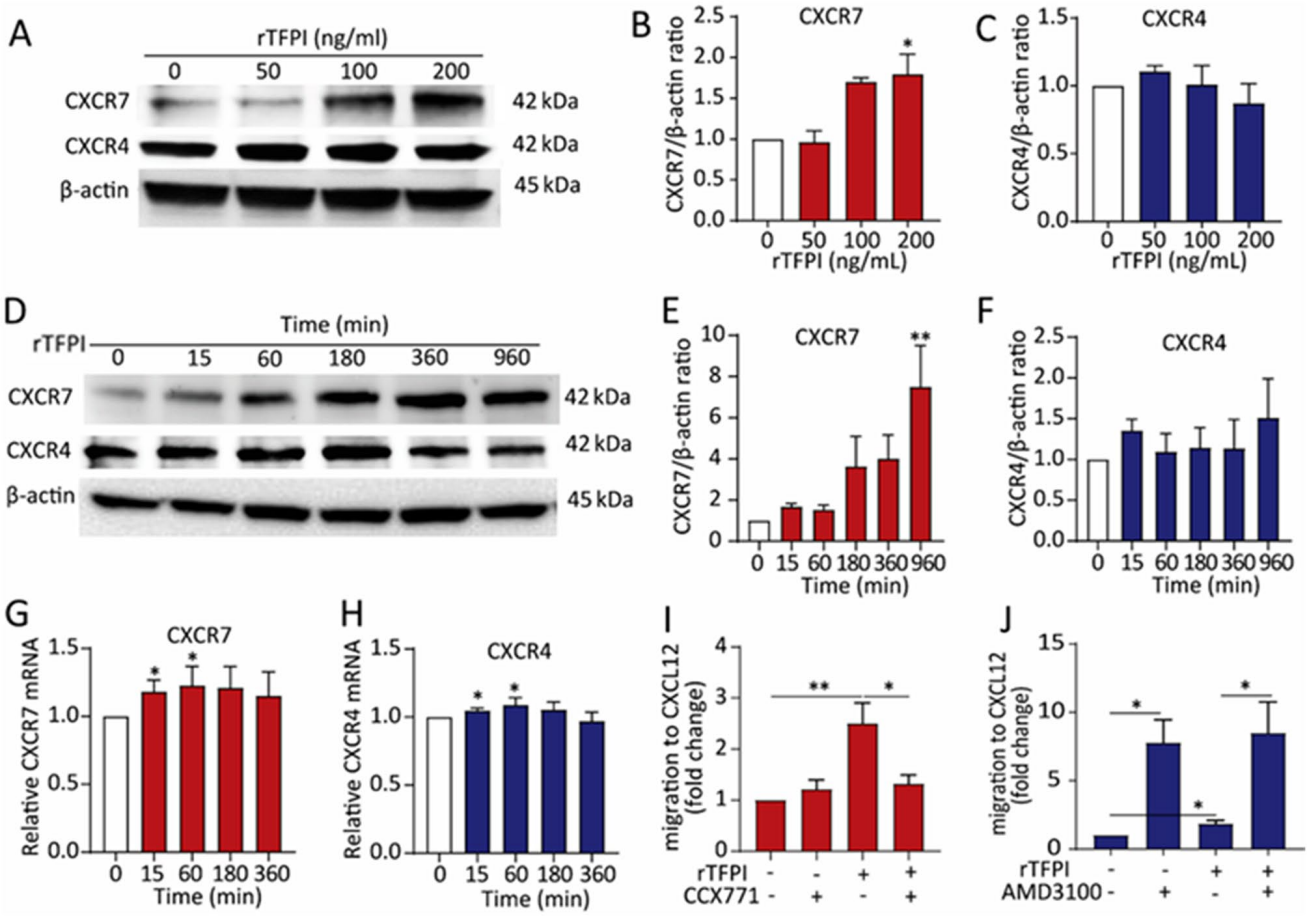
CXCR7 plays a key role in the effect of TFPI on the CXCL12-mediated TEM of CLL cells. (**A**) Western blotting and (**B**, **C**) quantification of CXCR7 and CXCR4 expression, relative to β-actin, after treating CLL cells with different doses of rTFPI for 24 h. n = 3 individuals, *P < 0.05. (**D**) Western blotting and (**E**, **F**) quantification of CXCR7 and CXCR4 expression, relative to β-actin, after treating the CLL cells with 200 ng/mL rTFPI for 0, 15, 60, 180, 360 and 960 min. n = 4 individuals, **P < 0.01. (**G**, **H**) mRNA expression of CXCR7 and CXCR4 was detected by quantitative PCR, relative to TBP, after treating the CLL cells with 200 ng/mL rTFPI for 0, 15, 60, 180, 360 and 960 min. n = 4 individuals; *P < 0.05. (**I**) After 24 h exposure of 200 ng/mL rTFPI, CLL cells were treated with 2 µM CCX771 for 1 h before they were used in the TEM assay. DMSO was used as control. n = 6 individuals; *P < 0.05, **P < 0.01. (**J**) After 24 h exposure of 200 ng/mL rTFPI, CLL cells were treated with 5 µg/mL AMD3100 for 1 h before they were applied to the transwells for TEM assay. For panels (**I, J**), results were presented as described in Fig. 1A. n = 6 individuals; *P < 0.05.

To understand how TFPI affects CXCL12-mediated TEM of CLL cells, we investigated the expression of CXCR4 and CXCR7 in the CLL cells. Western blots showed that after 24 h of rTFPI exposure, CXCR7 expression was dose-dependently upregulated in the CLL cells, while CXCR4 expression showed no increase (Fig. 3A–C). Flow cytometry analysis showed that surface expression of CXCR7 was increase by 24 h treatment of rTFPI (Supplementary Fig. 8). Moreover, Western blots showed that the expression of CXCR7 was increased over time after rTFPI exposure, but no obvious change in CXCR4 expression was observed (Fig. 3D–F). Quantitative RT-PCR showed a 16% increase of CXCR7 mRNA level 15 min after rTFPI exposure, but only a 4% increase of CXCR4 mRNA (Fig. 3G,H). To further address the involvement of CXCR7 in the TFPI-mediated TEM, CCX771^31^, a small molecule with high affinity and selectivity for CXCR7 was used. In the presence of CCX771, the enhanced TEM induced by rTFPI was strongly repressed, while CCX771 alone did not repress the migration potential of CLL cells (Fig. 3I). Surprisingly, the CXCR4 inhibitor AMD3100^32^ strongly increased CXCL12-mediated TEM both with and without TFPI (Fig. 3J). We concluded that CXCR7, but not CXCR4 is involved in the effect of TFPI on the TEM of CLL cells.

### The binding of TFPI to GPC3 on the surface of CLL cells was involved in the TFPI-mediated TEM

**Figure 4:**
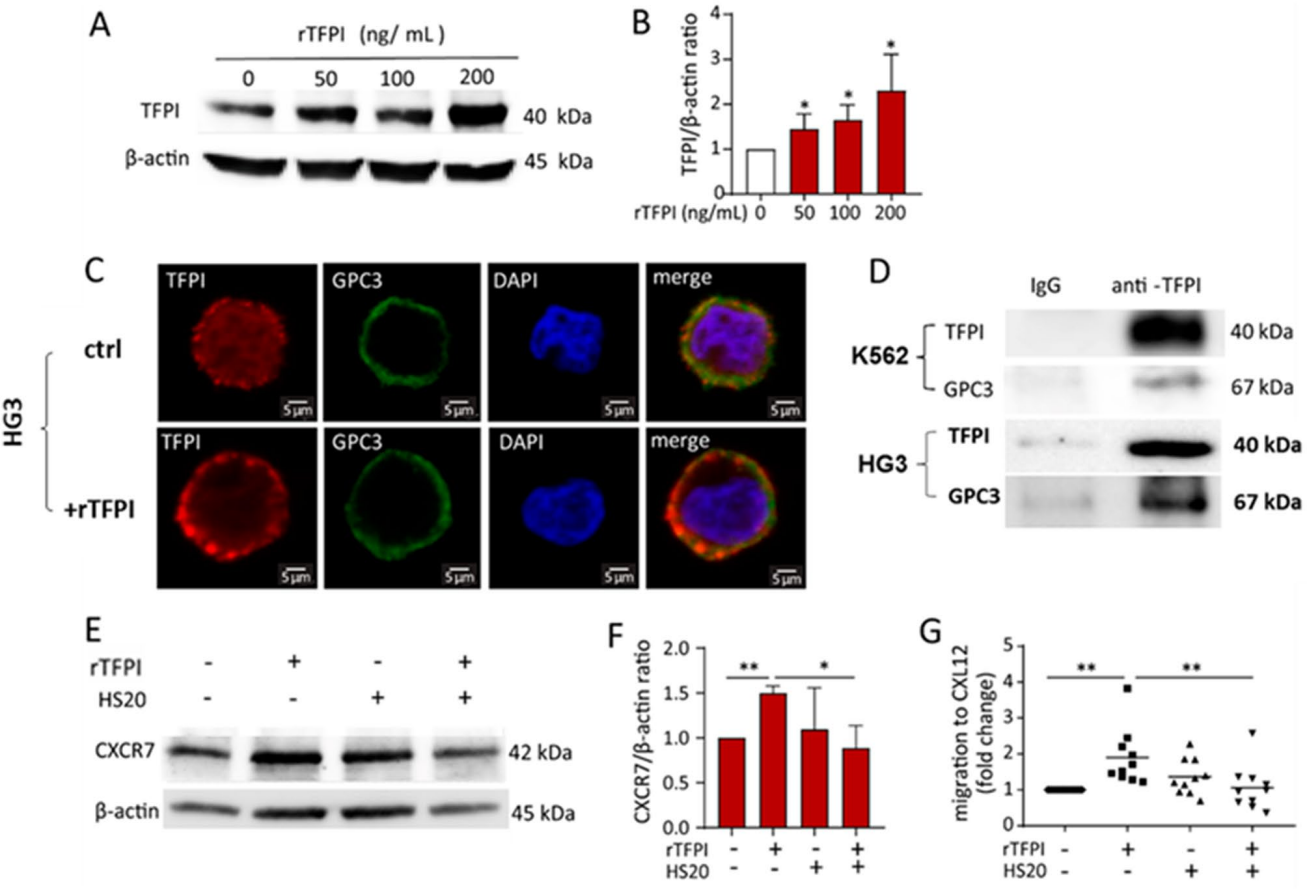
TFPI binds to GPC3 on the membrane of CLL cells. (**A**) Western blotting and (**B**) quantification of TFPI protein in the cell lysates of CLL patients, relative to β-actin, after 0, 50, 100 and 200 ng/mL rTFPI treatment. n = 3 individuals; *P < 0.05. (**C**) After 24 h treatment of 200 ng/mL rTFPI, co-localization of TFPI (red) and GPC3 (green) on the surface of human CLL cell line HG3 was assessed by immunofluorescence staining. Nuclei were stained with DAPI (blue). (**D**) Co-IP was performed in the HG3 and K562 cells. Immunoblots showed the bait protein TFPI and the pull-down of the prey protein GPC3. (**E**) Western blotting and (**F**) quantification of CXCR7 expression, relative to β-actin, after CLL cells were pre-treated with GPC3 antibody HS20 (100 µg/mL) for 1 h prior to 24 h treatment of 200 ng/mL rTFPI. Human IgG was used as control since HS20 is isolated from human serum. n = 4 individuals; *P < 0.05, **P < 0.01. (**G**) After CLL cells were pre-treated with HS20 before rTFPI treatment, the cells were washed and applied to TEM assay. Human IgG was used as control. Results were presented as we described in Fig. 2A. n = 10 individuals; **P < 0.01.

Western blot analyses showed a dose-dependent accumulation of TFPI protein in the cell lysates after rTFPI treatment for 24 h (Fig. 4A,B), which indicates that exogenous TFPI binds to or enters the CLL cells. Therefore, we hypothesized that a potential receptor for TFPI exists on the surface of CLL cells. Since TFPI is known to bind to cell membrane heparan sulphate proteoglycans, such as GPC3^20,33^, we examined the expression of GPC3 in CLL cells by immunofluorescence. Immunostaining showed that TFPI and GPC3 were co-expressed on the surface of the CLL cell line HG3 and stronger TFPI staining on the cell surface was detected after adding exogenous rTFPI (Fig. 4C). Co-IP experiments were performed to investigate the binding between TFPI and GPC3 in the CLL cell line HG3 and the myeloid leukemic cell line K562. The antibody against TFPI was used to precipitate the protein complex, and then immunoblots were performed to detect GPC3 protein. Results showed the co-precipitation of GPC3 along with TFPI in both HG3 and K562 cells (Fig. 4D). To assess whether GPC3 is involved in the effect of TFPI on regulating CXCR7 expression and increasing CXCL12-mediated TEM, HS20, a specific antibody against the heparan sulphate chains of GPC3, was applied to block the binding of TFPI to GPC3. Western blotting showed that rTFPI treatment increased the expression of CXCR7 and that pre-treatment with HS20 abolished the effect of TFPI, while HS20 itself did not affect CXCR7 expression (Fig. 4E,F). Furthermore, TEM results showed that HS20 pre-treatment abolished the effect of TFPI on TEM (p = 0.0074), although HS20 itself has no significant effect on the migration of CLL cells (p = 0.5) (Fig. 4G). We also analyzed the effect of addition of TFPI to HS20 compared to the effect of HS20 alone, however, no significant difference was found (p = 0.65).

### Activation of β-catenin was involved in the TFPI-mediated TEM

**Figure 5:**
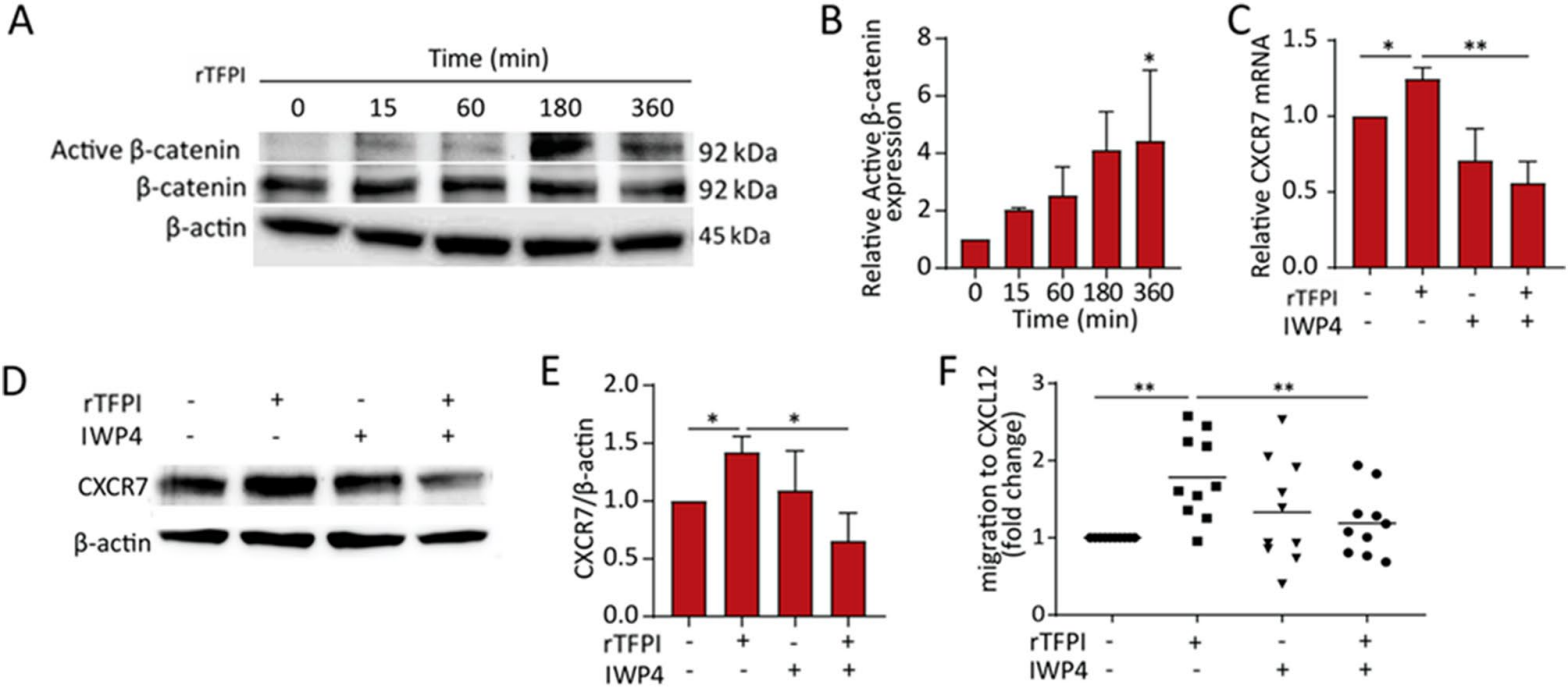
Activation of β-catenin is involved in the effect of TFPI on the expression of CXCR7 and the CXCL12-mediated TEM of CLL cells. (**A**) Western blotting and (**B**) quantification of the active β-catenin expression, relative to β-actin, after treating the CLL cells from patients with 200 ng/mL rTFPI for 0, 15, 60, 180 and 360 min. n = 3 individuals; *P < 0.05 (**C**) Quantitative PCR was used to detect the mRNA expression of CXCR7, relative to TBP, after CLL cells were pre-treated with IWP4 (5 µM) for 30 min prior to 24 h treatment of 200 ng/mL rTFPI, with DMSO as a control. n = 4 individuals; *P < 0.05, **P < 0.01. (**D**) Western blotting and (**E**) quantification of CXCR7 expression, relative to β-actin, after CLL cells were pre-treated with 5 µM IWP4 for 30 min prior to 24 h treatment of 200 ng/mL rTFPI, with DMSO as a control. n = 3 individuals; *P < 0.05. (**F**) CLL cells were pre-treated with 5 µM IWP4 for 30 min prior to 24 h treatment of 200 ng/mL rTFPI, with DMSO as a control. Then CLL cells were washed and applied to TEM assay. TEM results were presented as we described in Fig. 2A. n = 10 individuals; **P < 0.01.

Since Wnt/β-catenin signaling is known to be regulated by GPC3^21,25,34^, we examined if the binding of TFPI to GPC3 activates the Wnt/β-catenin signaling pathway in CLL cells. Western blotting showed that active β-catenin was detected 15 min after rTFPI exposure and was further increased over time (Fig. 5A,B). To confirm the involvement of Wnt/β-catenin signaling in the effect of TFPI, we used IWP4, a potent inhibitor for the palmitoylation of Wnt proteins. Quantitative PCR showed that CXCR7 mRNA expression was increased by rTFPI and this effect was abolished by IWP4 (Fig. 5C). Consistently, Western blotting showed that the effect of TFPI on CXCR7 expression was repressed by IWP4 (Fig. 5D,E). TEM results showed that IWP4 alone did not affect the migration of CLL cells, but pre-treating the CLL cells with IWP4 prior to rTFPI exposure fully prevented the effect of rTFPI on CLL cell migration (Fig. 5F).

## Discussion

TFPI was previously thought to be limited to its key role in regulating coagulation, but our data suggest that TFPI is involved in the migration of CLL cells. We found that plasma TFPI concentrations in CLL patients were higher than in the healthy controls, with particularly high levels in Binet stage C. It cannot be explained by the increase of platelets which are the main source of TFPI in the blood cells because most of CLL patients with Binet stage C have thrombocytopenia. Then it suggests that patients with higher levels of TFPI are more prone to tissue infiltration of CLL. In this study, the low numbers of patients in different stages has been a limitation. However, in our in vitro TEM study, we found consistently that TFPI enhanced CXCL12-mediated cell migration through binding to GPC3, activating β-catenin, and finally upregulating CXCR7 expression in the CLL cells.

A previous study showed that TFPI improves the migration and homing of hematopoietic stem cells through TFPI-mediated inhibition of the cleavage of CXCL12 by CD26^20^. However, we observed that TFPI increases CLL cell migration by upregulating the expression of CXCR7, a receptor for CXCL12. Either one or both pathways may operate in a given cell depending on the cell type.

CXCR7 together with CXCR4 are the two CXCL12 receptors, which play critical roles in CXCL12-mediated cell migration. It is known that CXCR4 is highly expressed by CLL cells and is involved in their migration^35^. Previous studies showed that CXCR7 plays a role in the migration of U937 leukemic cells and CD34+ stem cells^36^. Our study showed that CXCR7 is also expressed by CLL cells along with CXCR4. Interestingly, despite the higher levels of CXCR4 in CLL cells^35^, we observed that CLL cells migrate with lower efficiency and potency to CXCL12 than normal B cells, which is consistent with the previous studies^37–39^. Even though CXCR7 may be physically associated with CXCR4^40^, CXCR7 can independently induce cell signaling, provide survival advantage and participate in CXCL12-mediated migration^41,42^. In our study, the expression of CXCR7, not CXCR4, was upregulated by TFPI in CLL cells and thus enhanced the CXCL12-mediated cell migration. Our results are consistent with the study of hematopoietic stem cells which showed that the expression of CXCR4 is not affected by TFPI^20^. Moreover, the inhibition of CXCR7 abolished the effect of TFPI on the cell migration, supporting that CXCL12/CXCR7 interaction plays an important role in the TFPI-induced migration of CLL cells. In contrast, the CXCR4 inhibitor AMD3100 increased the migration potential of CLL cells. This is probably because AMD3100 also improves the binding of CXCL12 to CXCR7^43^, which improves the cell migration. However, CXCR7 inhibition itself does not repress cell migration suggesting that in addition to CXCL12/CXCR7 chemotaxis, other mechanisms might be involved in the regulation of CLL cell migration.

In our study, pre-treatment of CLL cells with recombinant TFPI increased TFPI protein in the cell lysates, suggesting that exogenous TFPI probably binds to CLL cells. Heparan sulphate proteoglycans are implicated in the binding and internalization of recombinant forms of TFPI, depending on their C-terminal polybasic portions^44^. GPC3 is a family member of heparan sulphate proteoglycans^45^. We found the co-expression of TFPI and GPC3 on the surface of CLL cells and co-IP confirmed the binding between the two proteins, which is consistent with previous studies on the hepatocellular carcinoma cell line HepG2^33^ and hematopoietic stem cells^20^. Moreover, after adding exogenous TFPI to CLL cells, more TFPI protein was observed on the cell membrane, partially co-localized with GPC3, which indicates that exogenous TFPI from stromal cells may affect the cell function by binding to the membrane protein GPC3. The heparan sulphate chains on GPC3 are located close to the C terminus and the cell surface, which mediate the interaction of GPC3 with other cell membrane proteins^46^. Therefore it is suggested that TFPI probably binds to GPC3 through its heparan sulphate chains. This hypothesis was supported by the results showing that the GPC3 antibody HS20, which recognizes the heparan sulphate chains and inhibits the activity of GPC3^47^, abolished the effect of TFPI on the expression of CXCR7 and the migration of CLL cells.

Accumulating evidence indicates that GPC3 activates the canonical Wnt signaling pathway in hepatocellular carcinoma cells^21^. Our data showed that the binding of TFPI to GPC3 on the CLL cells activated β-catenin, which is a key mediator of the canonical Wnt signaling pathway^48^. Researchers has found that Wnt3, Wnt5b, Wnt6, Wnt10a, Wnt14, and Wnt16, as well as the Wnt receptor Fzd3, are highly expressed in CLL, compared with normal B cells, which suggests that the Wnt signaling pathway is active in CLL^49^ and contributes to the survival of CLL cells^50^. In our study, a potent inhibitor for the palmitoylation of Wnt proteins were used to block the activation of Wnt/β-catenin pathway and we found that the effect of TFPI on both CXCR7 expression and TEM were impaired. It suggests that the activation of canonical Wnt/β-catenin pathway is involved in the CLL cell migration.

In conclusion, our results indicate that TFPI may enhance the capacity of CLL cells to transmigrate through multiple vascular endothelial beds and potentially contribute to organ infiltration.

## Supplementary Information

Supplementary Information.
