# Supplementary material for: Tissue factor pathway inhibitor upregulates CXCR7 expression and enhances CXCL12-mediated migration in chronic lymphocytic leukemia
Source: Sci Rep. 2021 Mar 4;11:5127. doi: 10.1038/s41598-021-84695-8 (PMC7933411; doi:10.1038/s41598-021-84695-8)
Supplement: Supplementary file 1 — Supplementary Information. [file 41598_2021_84695_MOESM1_ESM.pdf]

Tissue factor pathway inhibitor upregulates CXCR7 expression and enhances CXCL12-mediated migration in chronic lymphocytic leukemia

Xue Yan Cui[1-4*], Geir Erland Tjønnfjord[2,4,5], Sandip M. Kanse[6], Anders Erik Astrup Dahm[4,7], Nina Iversen[8], Christiane Filion Myklebust[2,3], Ling Sun[1], Zhong Xing Jiang[1], Thor Ueland[3], James J. Campbell[9], Mitchell Ho[10] and Per Morten Sandset[1-4*]

Supplementary figure 1 (corresponding to Figure 3A in the main manuscript)

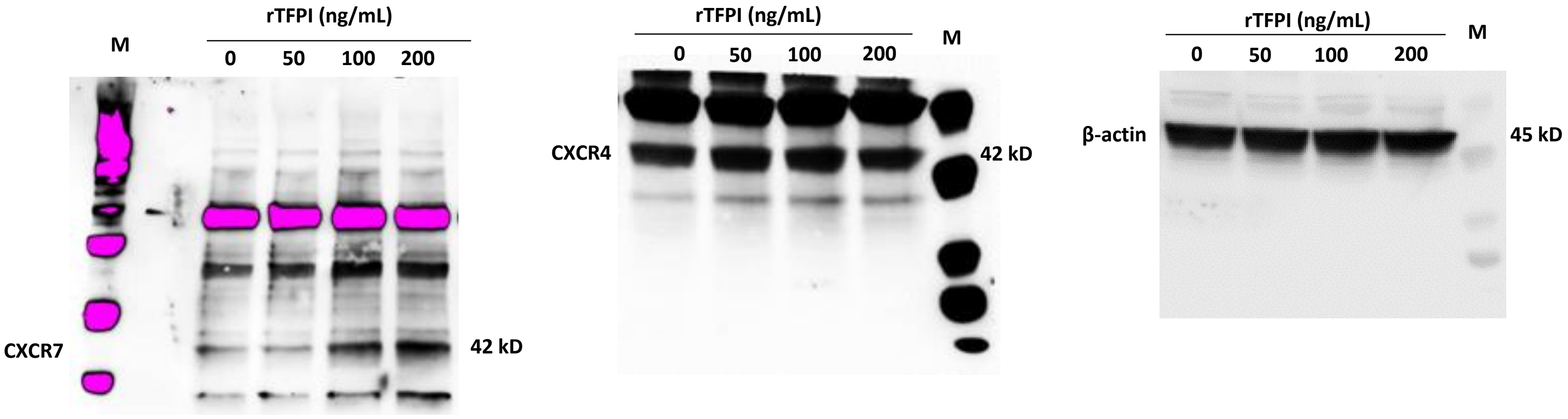

Fig. 3A Western blotting of CXCR7 and CXCR4 expression, relative to β-actin, after treating CLL cells with different doses of rTFPI for 24 h. n=3 individuals, *P<0.05. M= MW marker.

Supplementary figure 2 (corresponding to Figure 3D in the main manuscript)

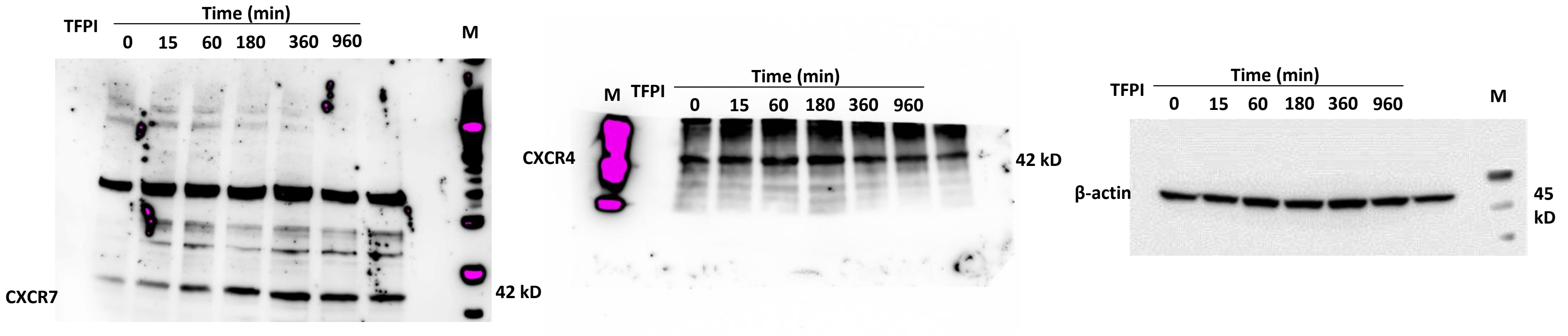

Fig. 3D Western blotting of CXCR7 and CXCR4 expression, relative to β-actin, after treating the CLL cells with 200 ng/mL rTFPI for 0, 15, 60, 180, 360 and 960 min. n=4 individuals, **P<0.01. M= MW marker.

Supplementary figure 3 (corresponding to Figure 4A in the main manuscript)

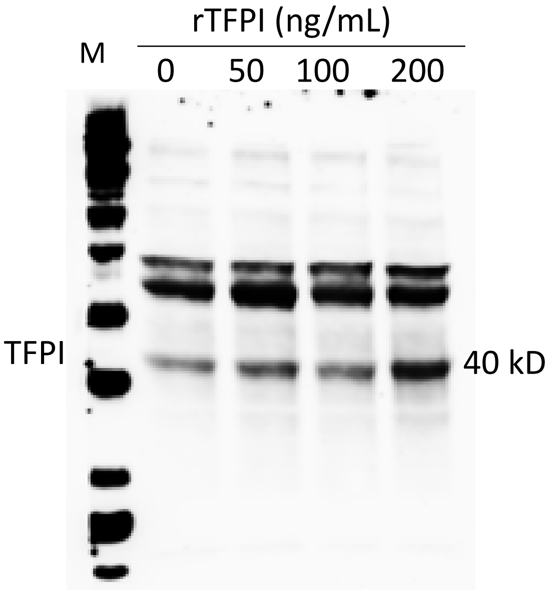

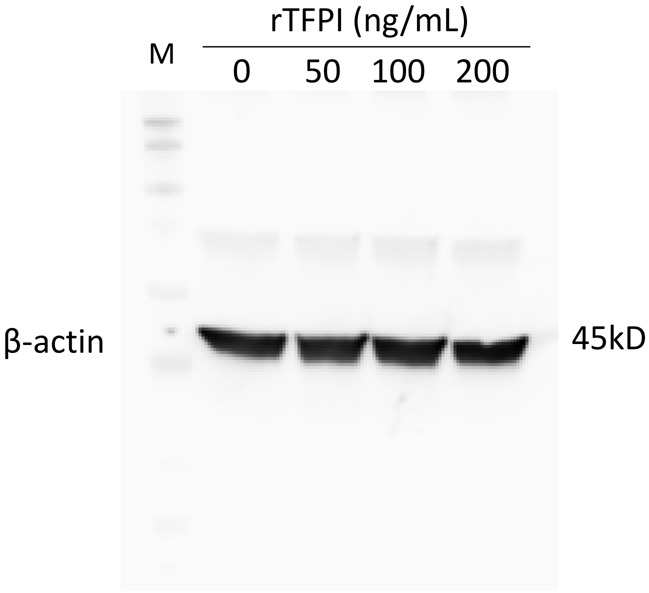

Fig. 4A Western blotting of TFPI protein in the cell lysates of CLL patients, relative to β-actin, after 0, 50, 100 and 200 ng/mL rTFPI treatment. n=3 individuals; *P<0.05. M= MW marker.

Supplementary figure 4 (corresponding to Figure 4D in the main manuscript)

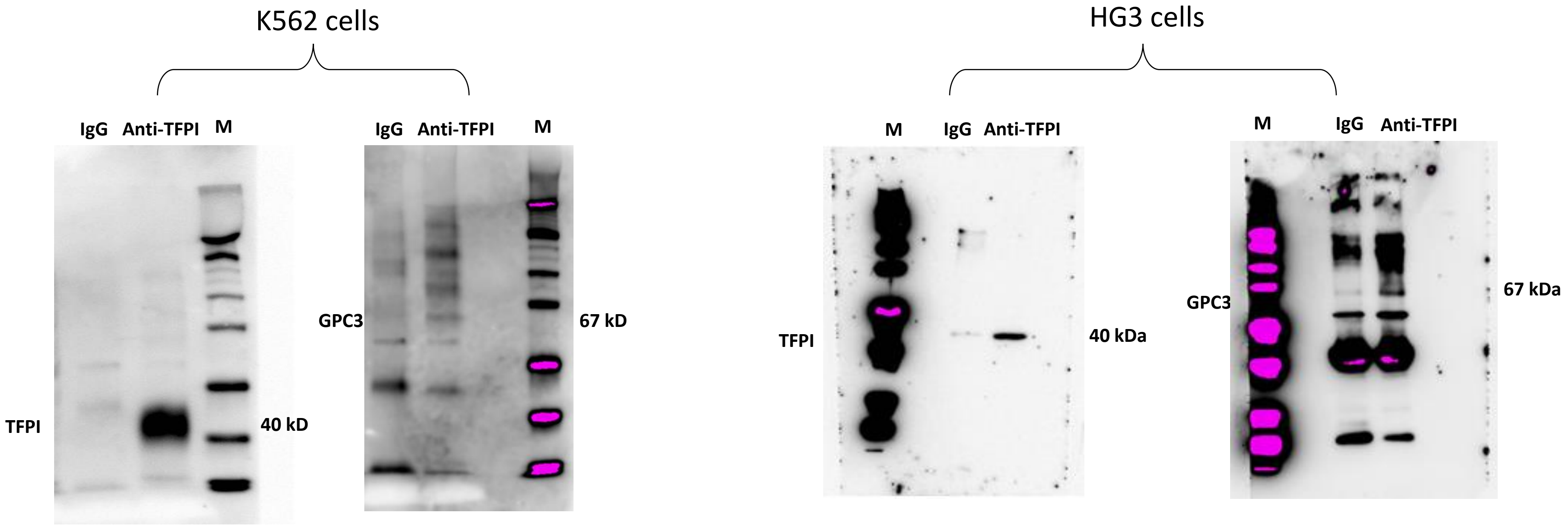

Fig. 4D Co-IP was performed in the HG3 and K562 cells. Immunoblots showed the bait protein TFPI and the pull-down of the prey protein GPC3. M= MW marker.

Supplementary figure 5 (corresponding to Figure 4E in the main manuscript)

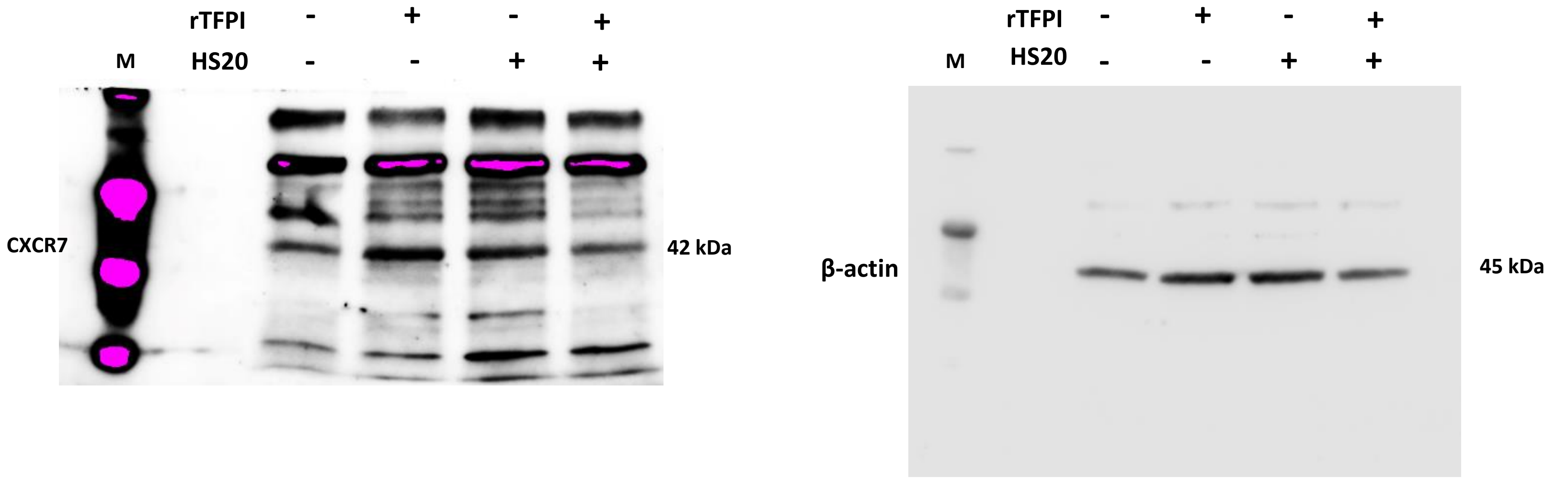

Fig. 4E Western blotting of CXCR7 expression, relative to β-actin, after CLL cells were pre-treated with GPC3 antibody HS20 (100 µg/mL) for 1 h prior to 24 h treatment of 200 ng/mL rTFPI. Human IgG was used as control since HS20 is isolated from human serum. n=4 individuals; *P<0.05, **P<0.01. M= MW marker.

Supplementary figure 6 (corresponding to Figure 5A in the main manuscript)

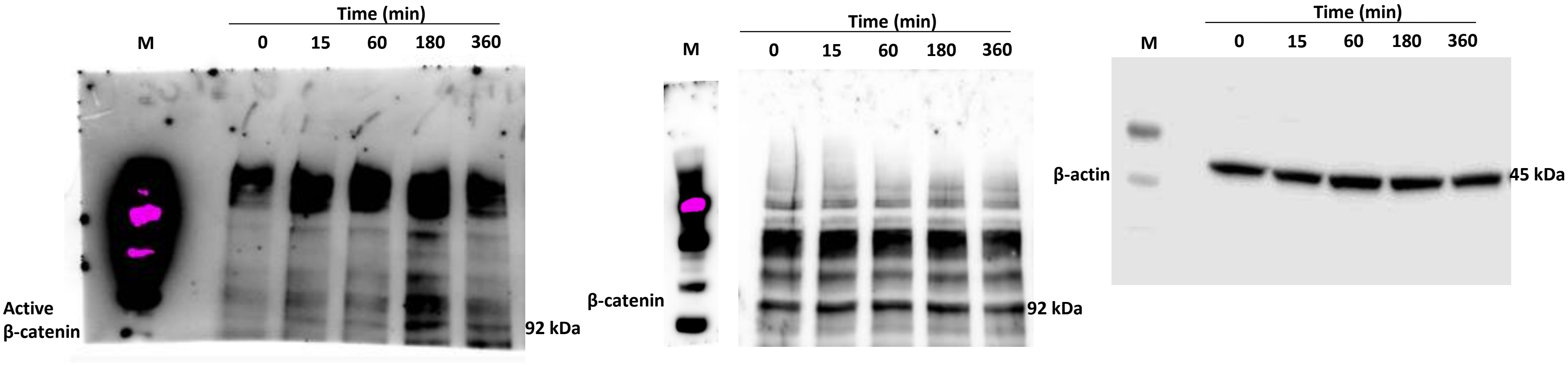

Fig. 5A Western blotting of the active β-catenin expression, relative to β-actin, after treating the CLL cells from patients with 200 ng/mL rTFPI for 0, 15, 60, 180 and 360 min. n=3 individuals; *P<0.05. M= MW marker.

Supplementary figure 7 (corresponding to Figure 5D in the main manuscript)

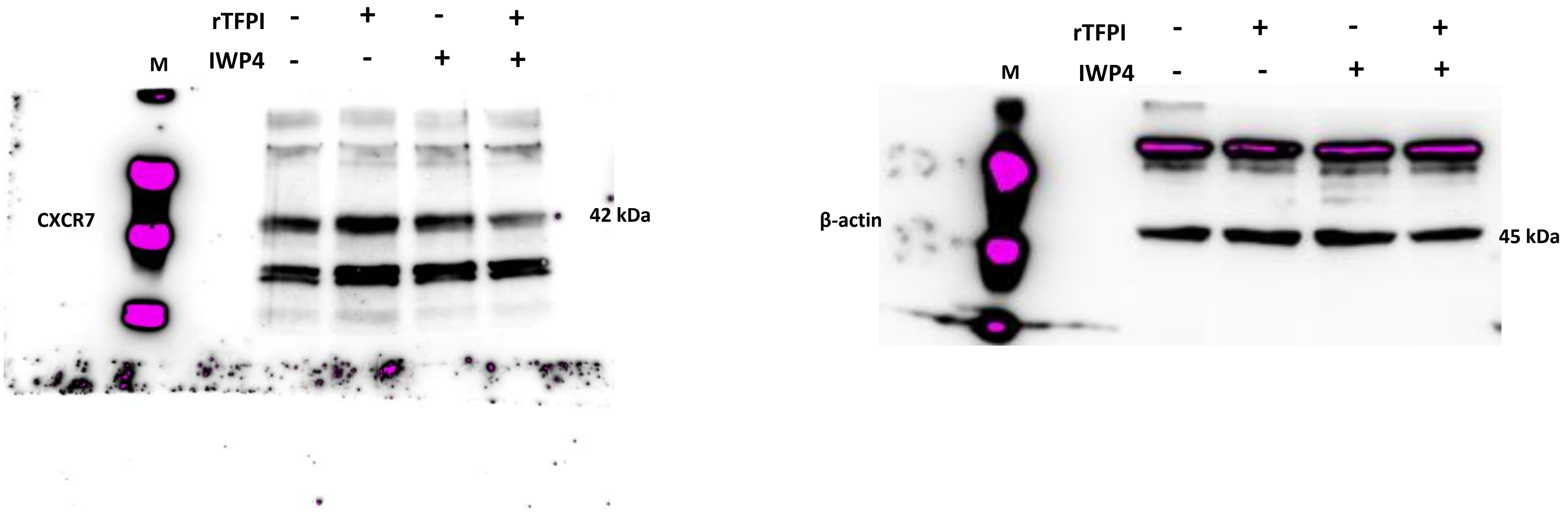

Fig. 5D Western blotting of CXCR7 expression, relative to β-actin, after CLL cells were pre-treated with 5μM IWP4 for 30 min prior to 24 h treatment of 200ng/mL rTFPI, with DMSO as a control. n=3 individuals; *P<0.05. M= MW marker.

Supplementary figure 8

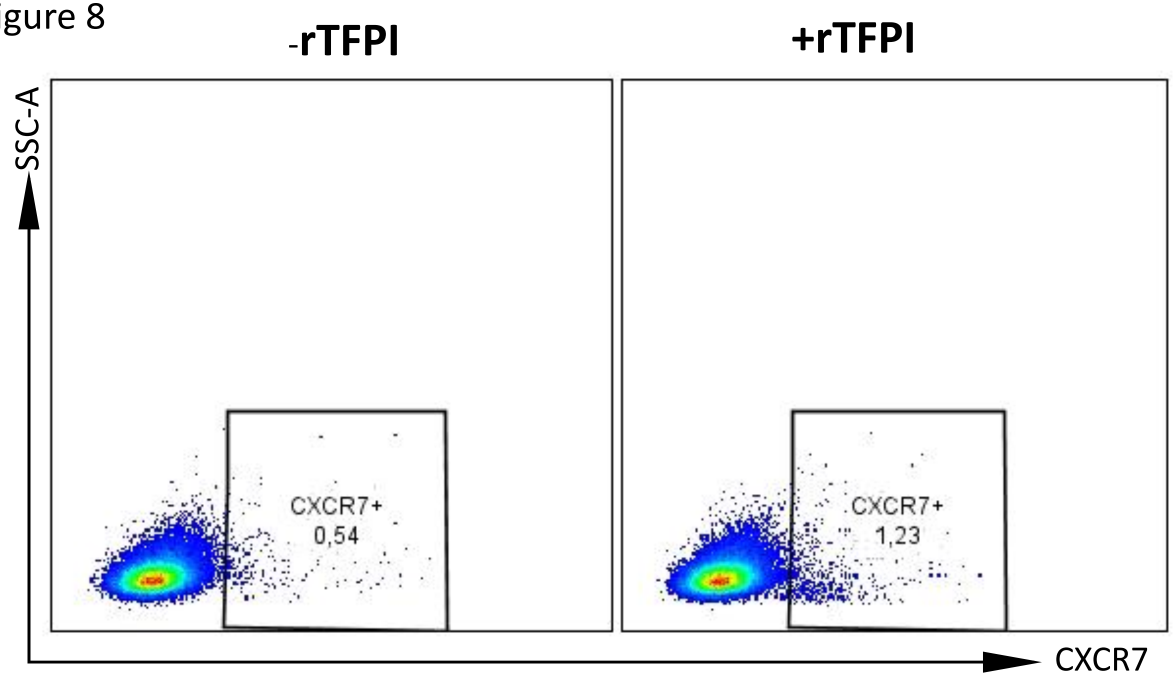

Supplementary figure 8. **Surface expression of CXCR7 was increased by TFPI.** CLL cells were isolated from a CLL patient (Binet stage C) and treated with 200ng/mL rTFPI for 24 h. After washing, the cells were blocked by human FcR blocking reagent (Miltenyi Biotec, Bergisch Gladbach, Germany) for 10 min at room temperature before they were stained with PE-conjugated anti-human CXCR7 antibody (Biolegend, San Diego, CA) for 1h. PE Mouse IgG2b was used as isotype control (Biolegend). CXCR7 expression was measured by a FACS Calibur flow cytometry (Becton Dickinson, Franklin Lakes, NJ, USA) and analyses were carried out using FlowJo software (Becton Dickinson). n=2 individuals.

Supplementary figure 9

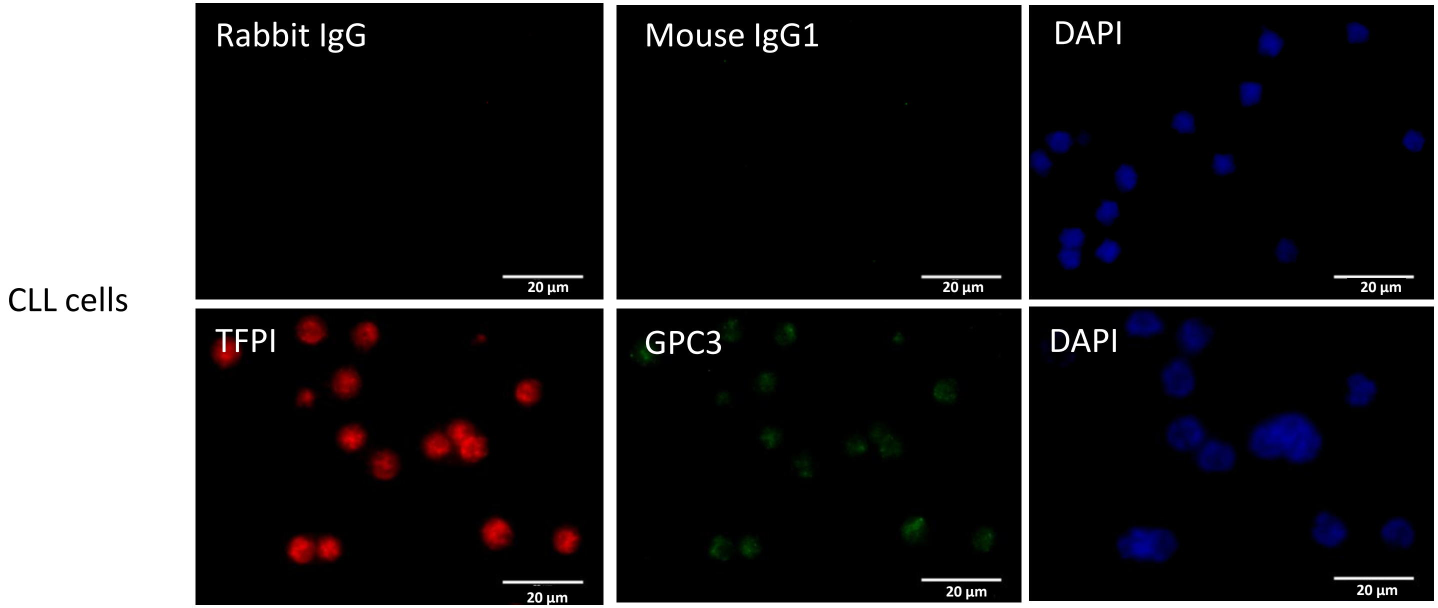

Supplementary figure 9. **Immunofluorescence staining of TFPI and GPC3 in the CLL cells from a patient.** Normal rabbit IgG and mouse IgG1 were used as isotype controls.
